# A Chimeric Affinity Tag for Efficient Expression and Chromatographic Purification of Heterologous Proteins from Plants

**DOI:** 10.3389/fpls.2016.00141

**Published:** 2016-02-15

**Authors:** Frank Sainsbury, Philippe V. Jutras, Juan Vorster, Marie-Claire Goulet, Dominique Michaud

**Affiliations:** ^1^Département de Phytologie–Centre de Recherche et d’Innovation sur les Végétaux, Université Laval, QuébecQC, Canada; ^2^Centre for Biomolecular Engineering, Australian Institute for Bioengineering and Nanotechnology, The University of Queensland, BrisbaneQLD, Australia; ^3^Department of Plant Production and Soil Science, Forestry and Agricultural Biotechnology Institute, University of PretoriaPretoria, South Africa

**Keywords:** plant molecular farming, protein purification, immobilized metal affinity chromatography, tomato cystatin SlCYS8, human α_1_-antitrypsin

## Abstract

The use of plants as expression hosts for recombinant proteins is an increasingly attractive option for the production of complex and challenging biopharmaceuticals. Tools are needed at present to marry recent developments in high-yielding gene vectors for heterologous expression with routine protein purification techniques. In this study, we designed the Cysta-tag, a new purification tag for immobilized metal affinity chromatography (IMAC) of plant-made proteins based on the protein-stabilizing fusion partner SlCYS8. We show that the Cysta-tag may be used to readily purify proteins under native conditions, and then be removed enzymatically to isolate the protein of interest. We also show that commonly used protease recognition sites for linking purification tags are differentially stable in leaves of the commonly used expression host *Nicotiana benthamiana*, with those linkers susceptible to cysteine proteases being less stable then serine protease-cleavable linkers. As an example, we describe a Cysta-tag experimental scheme for the one-step purification of a clinically useful protein, human α_1_-antitrypsin, transiently expressed in *N. benthamiana*. With potential applicability to the variety of chromatography formats commercially available for IMAC-based protein purification, the Cysta-tag provides a convenient means for the efficient and cost-effective purification of recombinant proteins from plant tissues.

## Introduction

As the practice of using plants to produce recombinant proteins matures in both industrial and academic contexts (Davies, 2010; Paul and Ma, 2011; Xu et al., 2012), the development of bespoke tags for protein purification has now become of particular relevance (Buyel et al., 2015). Fusion protein tags can permit effective recovery of high purity recombinant proteins from both prokaryotic and eukaryotic expression hosts (Pina et al., 2014), and even improve the stability and solubility of some labile or ‘difficult to express’ proteins (Terpe, 2003). A current trend in plant-based protein expression is the use of polypeptides, such as hydrophobins (Joensuu et al., 2010), elastin-like polypeptides (Conley et al., 2009; Floss et al., 2010) and the γ-zein motif ZERA (Torrent et al., 2009), that allow for the stabilization and non-chromatographic purification of recombinant protein fusion partners by the induction of insoluble aggregates. Fusion tags for the stabilization and chromatographic purification of recombinant proteins have also been devised, generally involving biologically active antibody fragments as affinity ligands. An example was provided by Obregon et al. (2006), who showed the α2 and α3 constant regions of a human IgG α-chain fusion partner to increase the accumulation of human immunodeficiency virus-p24 antigen by 13-fold in transgenic tobacco leaves and to allow for its affinity purification with anti-human IgG antisera. More recently, human IgG Fc fragments were used to improve the yields of anthrax toxin receptor (Andrianov et al., 2010) and camelid Nanobodies^®^ (De Buck et al., 2013) in transgenic plants, and to allow for the one-step purification of these proteins by protein A affinity chromatography.

Our general objective in this study was to engineer a widely applicable affinity chromatography tag for which non-biological –and cheap– affinity ligands are readily available in a variety of formats. Metal-chelating tags such as the popular poly-histidine (poly-His) tag have proved useful over the years to purify recombinant proteins from a variety of expression hosts by immobilized metal affinity chromatography (IMAC). Poly-His tags grafted at the C- or N-terminus of recombinant proteins have notably been used as purification ligands for protein recovery from different plant tissues (e.g., Leelavathi and Reddy, 2003; Valdez-Ortiz et al., 2005; Vardakou et al., 2012; de Souza et al., 2014), protocols and reagents for IMAC are available from numerous commercial suppliers, and IMAC procedures represent a generally convenient and cost-effective approach for the affinity purification of recombinant proteins at a lab scale (Lichty et al., 2005; Saraswat et al., 2013; Pina et al., 2014). Poly-His tags, however, may negatively affect the expression or activity of certain proteins (Woestenenk et al., 2004; Chant et al., 2005; Amor-Mahjoub et al., 2006; Renzi et al., 2006; Horchani et al., 2009; Sainsbury et al., 2009) and may sometimes be ineffective in native conditions due to their small size and variable accessibility at protein termini (Eschenfeldt et al., 2010). IMAC can be performed under denaturing conditions to make the poly-His motif more accessible (Terpe, 2003), but this is not applicable to those numerous proteins that cannot tolerate denaturation. Likewise, enzymatic procedures –such as the TAGZyme™ system– have been devised for the post-IMAC removal of His tag motifs (Pedersen et al., 1999; Schäfer et al., 2002), but the use of such systems remains costly and hardly accessible to most laboratories (Arnau et al., 2006; Waugh, 2011).

With these limitations in mind, we developed a chimeric poly-His tag, the ‘Cysta-tag,’ based on our recent observation that translational fusion to tomato multicystatin domain SlCYS8 can sustain, and even enhance, recombinant protein accumulation in leaves of the widely used expression host *Nicotiana benthamiana* (Sainsbury et al., 2013; Robert et al., 2016). We show the insertion of a poly-His motif in a solvent-exposed loop of SlCYS8 to produce an effective tag for IMAC purification of human α_1_-antitrypsin (α_1_AT), an anti-inflammatory protein with potential for the augmentation therapy of emphysema and other chronic obstructive pulmonary diseases (Stockley, 2015). We also document the variable stability of common protease cleavage sites for His tag removal, in the specific context of fusion proteins transiently expressed in *N. benthamiana*.

## Materials and Methods

### Structural Analyses

Structural models were generated *in silico* for SlCYS8 (GenBank accession no. AF198390) and tentative Cysta-tag hybrids to predict the impact of inserting a (His)_6_ [or 6x His] hexapeptide motif in the original cystatin structure. Twenty possible models were built for each possible variant using Modeller v. 9.7 (Eswar et al., 2006), with the NMR solution structure coordinates of oryzacystatin I (Nagata et al., 2000) as a template (Protein Data Bank accession no. 1EQK). The stereochemical quality of each model was assessed by comparison with the oryzacystatin structure using the PROCHECK program, v.3.5.4 (http://www.ebi.ac.uk/thornton-srv/ software/PROCHECK/; Laskowski et al., 1993). The best model for SlCYS8 and the best model for a tentative Cysta-tag variant were selected for visualization purposes, Cysta-tag engineering and heterologous expression in *Escherichia coli* or *N. benthamiana*.

### Gene Constructs and Cloning

An α_1_AT (SERPINA1; Accession No. NM_000295)-encoding DNA sequence was synthesized by GeneArt (Life Technologies) with an internal synonymous substitution in the original sequence to remove an undesired *Bsa*I restriction site. Sequences encoding SlCYS8, including a secreted version bearing the alfalfa protein disulphide isomerase (PDI) N-terminal signal peptide, were sourced from previously described constructs (Sainsbury et al., 2013). A (His)_6_ motif was introduced within SlCYS8 by extension overlap PCR between residues alanine (Ala)-62 and glycine (Gly)-63. Constructs for protein expression were assembled using a modified version of GoldenGate cloning (Engler et al., 2008), where counter selection against the *ccd*B gene yields only recombined expression plasmids (Sainsbury et al., 2012, 2013). Protein-encoding PCR fragments with appropriate GoldenGate recombination sites were blunt-end ligated into *Sma*I-digested pUC18strep in the presence of *Sma*I to limit plasmid self-ligation. Complementary oligonucleotides encoding various linker sequences with terminal extensions for recombination were annealed and similarly ligated into pUC18strep. For assembly into expression vectors, recombination reactions between expression plasmids and donor clones were driven by the simultaneous use of *Bsa*I and a T4-DNA ligase. Non-recombined pUC18strep and expression plasmid clones were eliminated by expression of the vector-selecting antibiotic or of the *ccd*B gene, respectively. For expression in *N. benthamiana*, a pEAQ plasmid (Sainsbury et al., 2009) modified to act as an acceptor plasmid for GoldenGate recombination was used (Sainsbury et al., 2013). For expression in *E. coli*, the expression vector pGEX-3X (GE Healthcare) was similarly modified to act as a GoldenGate acceptor (Sainsbury et al., 2012). To construct the green fluorescent protein (GFP) fusion, PCR fragments encoding GFP and the Cysta-tag with complementary overlaps of 20 nucleotides were assembled into pEAQ-*HT* (Sainsbury et al., 2009) linearized with *Age*I and *Stu*I, using the Gibson Assembly Master Mix (New England Biolabs). All constructs were verified by Sanger sequencing before heterologous protein expression.

### Cysteine Protease Inhibitory Activity

Bacterial expression and purification of recombinant cystatins were carried out as described previously (Sainsbury et al., 2013). Protein concentrations were determined by densitometry of Coomassie blue-stained gels after SDS-PAGE using the Phoretix 2-D Expression software, v. 2005 (Nonlinear Dynamics) and bovine serum albumin (Sigma–Aldrich) as a protein standard. Anti-papain activity of SlCYS8 and the Cysta-tag was determined by the monitoring of papain proteolysis progress curves with the fluorigenic synthetic peptide *Z*-Phe–Arg-methylcoumarin as a substrate, as described earlier (Goulet et al., 2008).

### Plant-Based Expression

pEAQ vectors for *in planta* expression were maintained in *Agrobacteria tumefaciens* strain AGL1 following transformation by electroporation. Bacterial cultures were first grown in lysis broth medium supplemented with appropriate antibiotics, and the bacteria then collected by gentle centrifugation. Bacterial pellets were resuspended in leaf infiltration medium (10 mM MES, pH 5.6, containing 10 mM MgCl_2_ and 100 μM acetosyringone) and incubated for 2–4 h at room temperature prior to transfection. Leaf infiltration was performed using a needle-less syringe as described earlier (D’Aoust et al., 2009), after mixing each protein-encoding (or empty vector) agrobacterial suspension with an equal volume of bacteria carrying the pEAQ express vector for transgene silencing suppression (Sainsbury et al., 2009). Infiltrated leaf tissue was collected 7 days post-infiltration for recombinant protein extraction and analysis, after incubating the plants at 23°C under a 16 h:8 h day–night photoperiod.

### Protein Extraction and Gel Electrophoresis

Leaf disks representing 160 mg of control (empty vector)-infiltrated tissue were harvested to determine protein expression rates following heterologous expression. The leaf disks were homogenized by disruption with a bead mill in three volumes of phosphate-buffered saline (PBS), pH 7.3, containing 5 mM EDTA, 0.05% (v/v) Triton X-100 (Sigma) and the cOMPLETE protease inhibitor cocktail for endogenous protease neutralization (Roche). Cell lysates were clarified by centrifugation at 20,000 × *g* for 5 min at 4°C and protein concentrations determined using the Bradford assay reagent (Thermo Scientific) with bovine serum albumin as a protein standard. Protein extracts were resolved by SDS-PAGE prior to Coomassie blue staining or immunodetection.

### Immunoblotting

Proteins for immunoblotting were resolved by 12% (w/v) SDS-PAGE and electrotransferred onto nitrocellulose sheets. Non-specific binding sites after electrotransfer were saturated with 5% (w/v) skim milk powder in PBS containing 0.025% (v/v) Tween-20, which also served as a dilution buffer for the antibodies. Human α_1_AT was detected with commercial polyclonal IgG raised in rabbit against this protein (US Biologicals) and alkaline phosphatase-conjugated goat anti-rabbit IgG secondary antibodies (Sigma–Aldrich). SlCYS8 and the Cysta-tag were detected with commissioned polyclonal IgG (Agrisera) raised in rabbit against a bacterially expressed SlCYS8 (Girard et al., 2007) and alkaline phosphatase-conjugated goat anti-rabbit IgG secondary antibodies (Sigma–Aldrich). The Cysta-tag was also detected with mouse anti-poly-His IgG (Cell Signaling Technologies) and horseradish peroxidase-conjugated IgG secondary antibodies (Sigma–Aldrich). GFP was detected with mouse anti-GFP antibodies (Cell Signaling Technologies) and horseradish peroxidase-conjugated secondary antibodies. Colorimetric signals for phosphatase-conjugated antibodies were developed with nitro blue tetrazolium chloride and 5-bromo-4-chloro-3-indolyl phosphate as a substrate (Sigma–Aldrich). Electrochemiluminescent signals for peroxidase-conjugated antibodies were generated with the Clarity Western ECL Substrate™ (Bio-Rad).

### Quantitative ELISA

Enzyme-linked immunosorbent assays (ELISA) were performed to quantify α_1_AT based on a procedure described earlier for human α_1_-antichymotrypsin (α_1_ACT; Sainsbury et al., 2013). Immulon 2HB ELISA plates (Thermo Scientific) were coated with duplicate samples of soluble protein extract diluted 1:50 to 27–30 μg/mL in PBS, pH 7.3. Non-specific binding sites were blocked with 1% (w/v) casein in PBS before application of anti-human α_1_AT diluted in PBS with 0.25% (w/v) casein. Anti-rabbit IgG conjugated to horseradish peroxidase were used as secondary antibodies, followed by colour signal development with the 3,3′,5,5′-tetramethylbenzidine SureBlue™ peroxidase substrate (KPL). The absorbance was read at 450 nm after adding 1 N HCl to stop color development. A standard curve was generated for each plate with human α_1_AT (EMD Chemicals) diluted in control extracts from tissue infiltrated with an empty vector, to account for possible matrix effects.

### Fusion Protein Purification

Cysta-tagged proteins were purified from crude protein extracts using the ÄKTA Prime Plus Liquid Chromatography System (GE Healthcare). Cysta-tagged α_1_AT was purified from 5 g of infiltrated tissue ground in liquid nitrogen and resuspended in three volumes of extraction buffer (20 mM sodium phosphate, pH 7.4, 0.5 M NaCl) containing EDTA-free cOMPLETE protease inhibitor cocktail (Roche). Leaf tissue expressing the Cysta-tag–GFP fusion was disrupted in three volumes of the same buffer using a PT1200 Polytron homogenizer (Kinematica). The leaf lysates were clarified by centrifugation at 20,000 × *g* for 10 min at 4°C, the supernatants frozen overnight at –80°C, and the mixtures centrifuged again to remove insoluble debris. Dithiothreitol (DTT; to 1 mM) and imidazole (to 20 mM) were added to the extracts, and the mixtures submitted to a final centrifugation round to remove precipitates. The resulting extracts were passed through a 0.45 μm syringe filter and approximately 12 ml was injected into a 5-ml sample loop in order to fill the loop completely. Samples were loaded onto 1-ml HisTrap columns for IMAC (GE Healthcare) and washed with extraction buffer containing 1 mM DTT and 20 mM imidazole. Immobilized proteins were eluted with 400 mM imidazole in extraction buffer containing 1 mM DTT, and the recovered fractions stored at –80°C or immediately prepared for SDS-PAGE. Yield and purity of the α_1_AT eluates were calculated relative to starting amount of α_1_AT and total protein content, respectively.

### Cysta-Tag Proteolytic Removal

Cysta-tag removal was done by protease treatment with the common linker processing enzyme human factor X_a_ (New England Biolabs). Purified protein samples were dialyzed overnight in 20 mM Tris-HCl, pH 8.0, containing 100 mM NaCl and 2 mM CaCl_2_. The Cysta-tag–protein (GFP) fusion was adjusted to a working concentration of 500 ng/μl and digested with factor X_a_ at molar ratios of 1:20 or 1:50. Protease reactions were performed at 20°C in total volumes of 100 μl. Samples were collected at different time points, and factor X_a_ activity stopped by the addition of SDS-PAGE sample loading buffer and heating for 5 min at 95°C.

## Results And Discussion

### Cysta-Tag Design, Structure, and Expression

**FIGURE 1:**
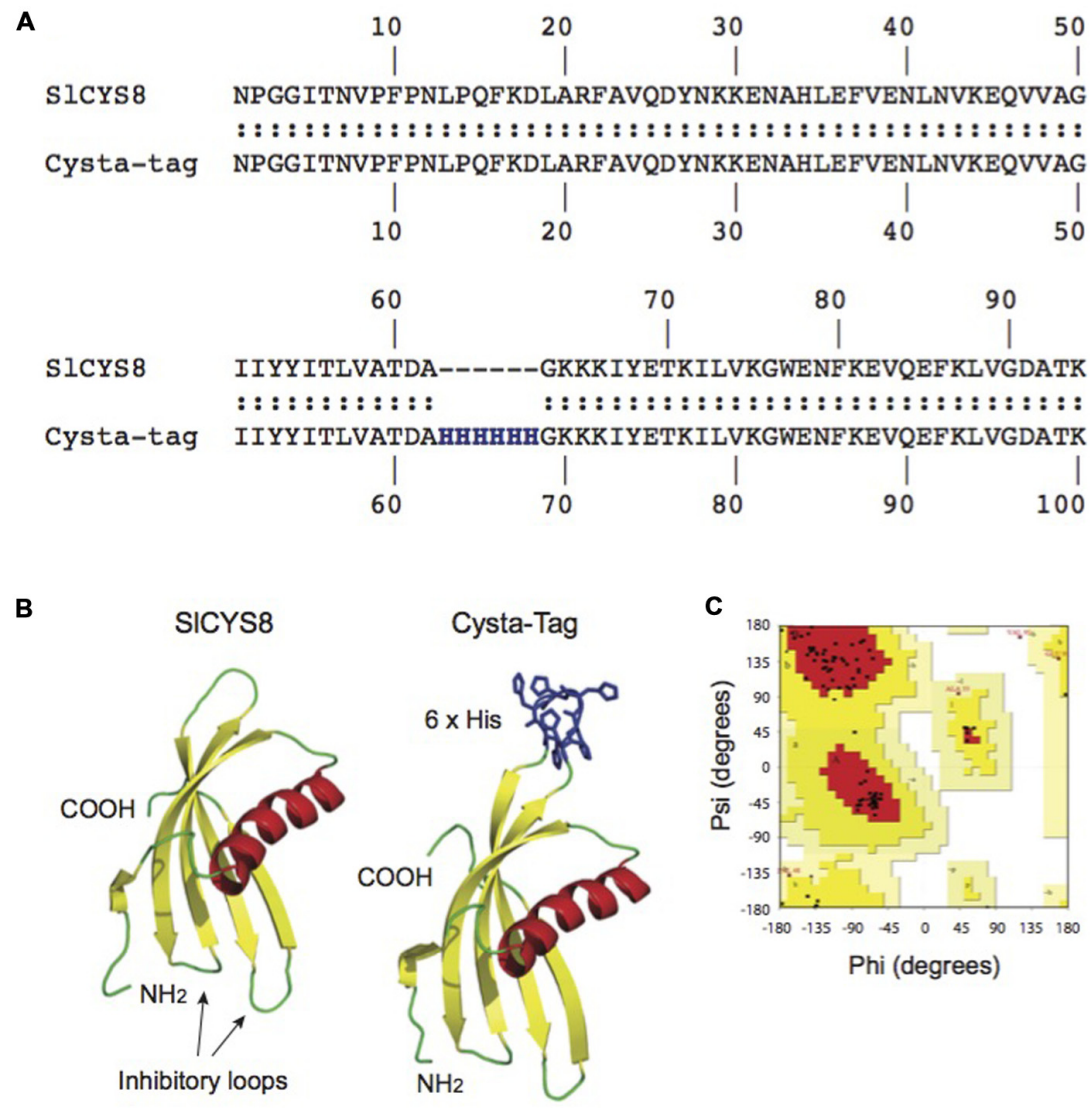
***In silico* modeling of the Cysta-tag.** Computationally modeling putative His-motif variants of SlCYS8 based on the NMR solution structure of oryzacystatin-I (PDB 1EQK) identified surface loop residues Ala-62 and Gly-63 as a possible target site for (His)_6_ motif insertion with minimal impact on the template structure **(A)**. Graphical representation of the *in silico* models shows location of the poly-His motif at this position, away from the N-terminus and inhibitory loops involved in protease inhibition **(B)**. A Ramachandran plot produced with PROCHECK for the ‘Ala-62–(His)_6_–Gly-63’ Cysta-tag model **(C)** shows more than 90% of the amino acid residues (black dots) to fall within the ‘most favored’ (red) and ‘additional allowed regions’ (yellow) areas of the graph, as expected for good quality structural models (Morris et al., 1992).

We took a rational approach to the design of the Cysta-tag, taking into account proximity of the poly-His tag insertion site to (1) the two inhibitory loops and N-terminal trunk of SlCYS8, which both contribute to the biological activity of the protein (Benchabane et al., 2010); and (2) the C-terminus of the cystatin, given the need to avoid steric interference from the fusion partner on IMAC substrate binding. Modeling attempts with these considerations in mind led us to select a structurally unconstrained site for the insertion of a (His)_6_ motif, between residues alanine (Ala)-62 and glycine (Gly)-63 (**Figure 1A**). *In silico* modeling of the chimeric protein resulted in a putative Cysta-tag variant with a predicted tertiary structure closely matching the tertiary structure of SlCYS8, aside from an extended surface loop with the poly-His motif away from the protease inhibitory loops (**Figure 1B**). Ramachandran plots were produced with the inferred structural coordinates of SlCYS8 (not shown) and the ‘Ala-62–(His)_6_–Gly-63’ Cysta-tag (**Figure 1C**) to confirm the stereochemical quality of our structural models (Laskowski et al., 1993). For both two proteins, more than 90% of the amino acid residues (black dots on **Figure 1C**) fell within the ‘most favored’ (red) and ‘additional allowed regions’ (yellow) areas of the graph, indicating adequate stereochemical quality of the predicted structures (Morris et al., 1992) and eventual robustness of the chimeric protein tag.

**FIGURE 2:**
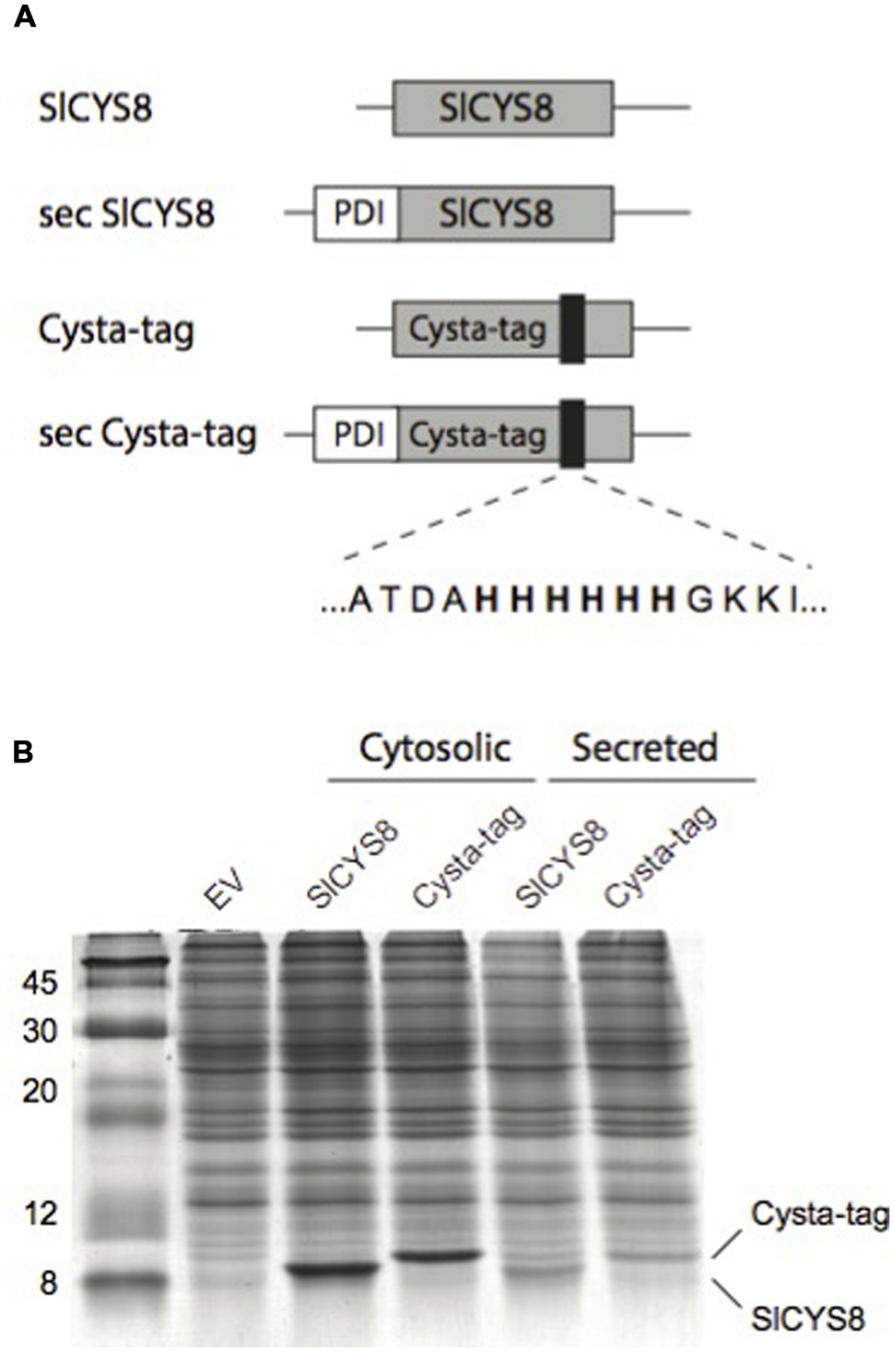
**Assembly and expression of the Cysta-tag in *N. benthamiana*. (A)** Schematic representation of gene constructs for expression of SlCYS8 and the Cysta-tag *in planta*, showing the relative location of poly-His motif (black areas) and flanking residues. PDI, signal peptide of alfalfa protein disulphide isomerase for targeting to the cell secretory pathway (sec). **(B)** Coomassie blue-stained polyacrylamide slab gel with protein extracts of leaf tissue expressing SlCYS8 and Cysta-tag in the cytosol or the secretory pathway. Protein bands for SlCYS8 and the Cysta-tag show similar steady-state levels in leaves and a slightly higher molecular weight for the Cysta-tag due to insertion of the (His)_6_ motif. EV stands for protein extract from leaf tissue infiltrated with agrobacteria harboring an ‘empty’ control vector.

We produced the –Ala-62–(His)_6_–Gly-63–Cysta-tag variant in *E. coli* to assess its overall stability and protease inhibitory activity compared to SlCYS8, using papain as a model target protease. Confirming a negligible impact for the inserted (His)_6_ motif on the cystatin template, an apparent dissociation constant of 42 nM was calculated for the Cysta-tag toward papain, similar to a dissociation constant of 43 nM determined for the original cystatin. Gene constructs were assembled to express SlCYS8 and the Cysta-tag in *N. benthamiana* leaves, either retained in the cytosol or targeted to the cell secretory pathway, to assess the impact of (His)_6_ motif insertion on stability of the cystatin *in planta* (**Figure 2A**). Coomassie blue-stained polyacrylamide slab gels following SDS-PAGE (**Figure 2B**) and immunoblots to confirm their identity (not shown) showed SlCYS8 and the Cysta-tag to accumulate at similar levels in both the cytosol and the apoplast, again indicating little impact of the poly-His motif on SlCYS8 overall stability and suggesting eventual robustness of the Cysta-tag as a fusion partner *in planta*.

### Expression of Cysta-Tag Fusions in *N. benthamiana*

**FIGURE 3:**
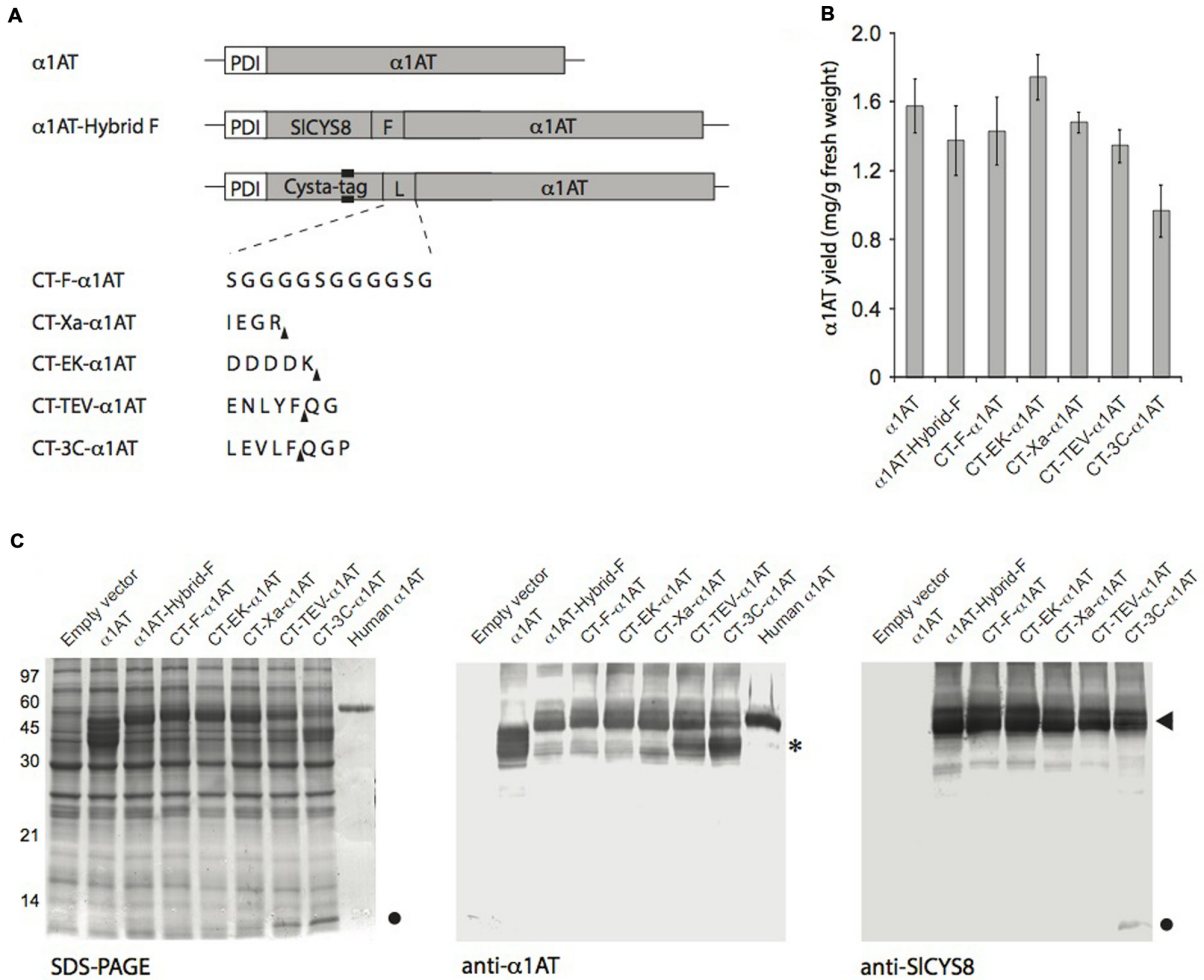
**Transient expression of Cysta-tag–α_1_AT fusions with different protease-cleavable linkers in *N. benthamiana* leaves. (A)** Schematic representation of the Cysta-tag (CT)–α_1_AT fusions, showing domain organization, location of the poly-His motif (black area) and identity of the inserted linker (L). F, flexible linker stable *in planta* (see Sainsbury et al., 2013); Xa, cleavage motif for human factor X_a_; EK, cleavage motif for bovine enterokinase; TEV, cleavage motif for *Tobacco etch virus* protease; 3C, cleavage motif for *Rhinovirus* 3C protease. Arrowheads in upright position show protease cleavage sites. **(B)** ELISA for quantitation of α_1_AT in crude protein extracts of leaves expressing secreted α_1_AT or the different Cysta-tag–α_1_AT fusions. Each bar is the mean of three replicate values ±SD. **(C)** SDS-PAGE separation of α_1_AT and Cysta-tag hybrids and their immunodetection with anti-α_1_AT and anti-SlCYS8 polyclonal antibodies. Asterisk (^∗^) on the right highlights free α_1_AT in leaf extract, arrowhead Cysta-tag–α_1_AT fusion protein, and closed circle free Cysta-tag. Human α_1_AT on right lanes of the Coomassie blue-stained gel and anti-α_1_AT immunoblot corresponds to a commercially available, highly glycosylated form of the protein.

We recently reported a strong positive effect of SlCYS8 used as a fusion partner on stability of an α_1_AT-related protein, α_1_ACT (Baker et al., 2007), in *N. benthamiana* leaves (Sainsbury et al., 2013). To demonstrate effectiveness of the Cysta-tag protein as a fusion partner moiety in plants, we chose to tag the clinically relevant α_1_AT, which has been the target for recombinant protein expression in plants where it can be produced in an active form (Terashima et al., 1999; Nadai et al., 2009; Zhang et al., 2012; Castilho et al., 2014). Because glycosylation of α_1_AT imparts increased stability to the normally secreted protein (Kwon and Yu, 1997), fusions were generated with a secreted version of the Cysta-tag (**Figure 3A**). For the gene constructs we used a mature form of α_1_AT lacking 23 amino acids at the N-terminus, as no well defined structure could be observed in this region by X-ray crystallography (Patschull et al., 2011) and because the corresponding N-terminal sequence in α_1_ACT, also presenting an undefined structure (Wei et al., 1994), was reported to undergo restricted proteolysis in a plant cell environment (Benchabane et al., 2009).

We fused α_1_AT to the Cysta-tag using the generic flexible peptide linker Gly_(4)_Ser, reported earlier to be proteolysis-resistant in the secretory pathway of *N. benthamiana* leaf cells (Sainsbury et al., 2013). Expressing un-tagged α_1_AT as well as fusions to either SlCYS8 or the Cysta-tag showed no positive effect of the plant cystatin on steady-state levels of secreted α_1_AT 7 days post-infiltration (**Figures 3B,C**), in sharp contrast with the positive effect reported earlier for SlCYS8 used as a fusion partner for α_1_ACT (Sainsbury et al., 2013). This, however, could be expected given the very high accumulation rate of more than 1.5 mg/g fresh weight tissue measured for α_1_AT (**Figure 3B**), much higher than the accumulation rate obtained for α_1_ACT expressed in the same expression system (Sainsbury et al., 2013). Most importantly, fusion to the Cysta-tag resulted in expression levels comparable to those observed with SlCYS8–α_1_AT (**Figures 3B,C**).

Since the Cysta-tag is biochemically active and because it could, with a molecular mass of ∼11 kDa, physically interfere with stability and activity of the fusion partner, we investigated the resistance of commonly used cleavable linkers to degradation by host plant endogenous proteases. To this end, we assembled Cysta-tag–α_1_AT fusions with cleavage motifs for two Ser proteases, bovine enterokinase and human factor X_a_, and for two Cys proteases, *Tobacco etch virus* (TEV) protease and *Rhinovirus* 3C protease (3C; **Figure 3A**). The four linkers showed variable stability *in planta*, with those acted on by Ser proteases being substantially more stable than those recognized by Cys proteases, which both led to an accumulation of free Cysta-tag detectable on Coomassie blue-stained gels (**Figure 3C**). In addition to being stable in the plant cell secretory pathway, the enterokinase and factor X_a_ cleavage motifs do not leave residual amino acids at the N-terminus of downstream-located proteins upon cleavage, unlike cleavage motifs of the TEV and 3C proteases expected to leave two or three non-native residues (see **Figure 3A** for expected cleavage sites). Our observations suggest that Ser protease-cleavable motifs such as those of enterokinase and factor X_a_ may be most useful in *N. benthamiana* expression platforms, both as stable linkers *in planta* before leaf processing and as convenient cleavable linkers for tag removal following recombinant protein purification.

### Purification of Plant-Made α_1_AT via the Cysta-Tag

**FIGURE 4:**
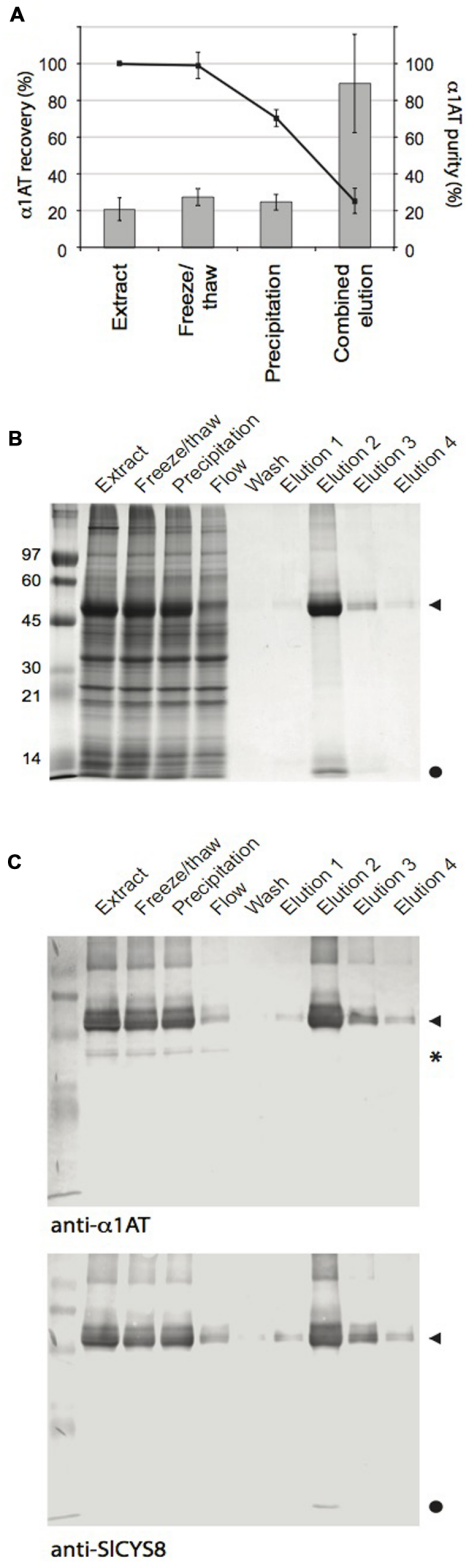
**IMAC purification of the Cysta-tag–α_**1**_AT fusion.** Cysta-tag and α_1_AT separated by the factor X_a_-cleavable sequence were transiently expressed in *N. benthamiana* leaves. **(A)** α_1_AT recovery (line) and purity (columns) rates during purification. Calculations were made based on ELISA assays for α_1_AT and leaf total soluble protein determinations. Data are mean values of three independent purification rounds ±SD. **(B)** Coomassie blue-stained polyacrylamide gels following SDS-PAGE showing key protein fractions during the purification process. Identity of the Cysta-tag–α_1_AT fusion was confirmed by immunodetections with anti-α_1_AT and anti-SlCYS8 primary antibodies **(C)**. Arrowheads point to the Cysta-tag–α_1_AT fusion, closed circles to free Cysta-tag and asterisk (^∗^) to free α_1_AT.

We submitted leaf tissue expressing Cysta-tag–α_1_AT to a routine IMAC procedure to confirm usefulness of the Cysta-tag as an affinity ligand for recovery of heterologous proteins in native conditions (**Figure 4**). A preliminary freeze/thaw treatment of clarified extracts resulted in the precipitation of a significant fraction of ribulose-1,5-*bis*phosphate carboxylase oxygenase (Rubisco), a major protein contaminant in crude leaf extracts (Robert et al., 2015), with no notable loss of the Cysta-tagged fusion (**Figure 4A**). Approximately 22% of total soluble proteins was lost during the process, compared to only a 1% average decrease in α_1_AT as measured by quantitative ELISA. Addition of imidazole to the protein extracts after freezing resulted in substantial protein precipitation leading to a further 18% loss of total protein and a concomitant 30% loss of α_1_AT relative to initial level in untreated extracts. These numbers represent an average across three independent purifications of factor X_a_-linked fusion protein. Similar rates were obtained with the enterokinase-linked Cysta-tag–α_1_AT fusion (Supplementary Figure S1), which suggests consistency of protein precipitation rates across leaf pre-purification steps and no significant impact of the cleavage linker on protein loss during early downstream processing.

Coomassie blue-stained gels and immunoblots confirmed that most of the Cysta-tag–α_1_AT fusion in clarified extracts was retained in HisTrap columns, while un-tagged α_1_AT did not bind in the presence of 20 mM imidazole (**Figure 4B**). During optimization of the elution conditions, we found that lower concentrations of imidazole resulted in non-specific binding of RuBisCO into the column, and higher concentrations to reduced retention of the Cysta-tag fusion. The 55-kDa protein fusion expected for Cysta-tagged α_1_AT was effectively eluted by the addition of 400 mM imidazole, to give final recovery yield and purity rate of about 25 and 90%, respectively, for both factor X_a_- and enterokinase-cleavable fusions (**Figure 4** and Supplementary Figure S1). Since starting amounts of α_1_AT in leaves were around 1.6 mg per g fresh weight (*see* **Figure 3B**), this represents a recovery rate for the expressed protein of ∼0.4 mg per g fresh weight, equivalent to about 5% of total extracted leaf protein. A protein contaminant likely corresponding to free Cysta-tag was sometimes visible in Coomassie blue-stained gels after purification, which could in theory be removed along with released Cysta-tag subsequent to proteolytic tag removal by further IMAC or other techniques such as ion exchange or size exclusion chromatography.

### Enzymatic Removal of the Cysta-Tag

**FIGURE 5:**
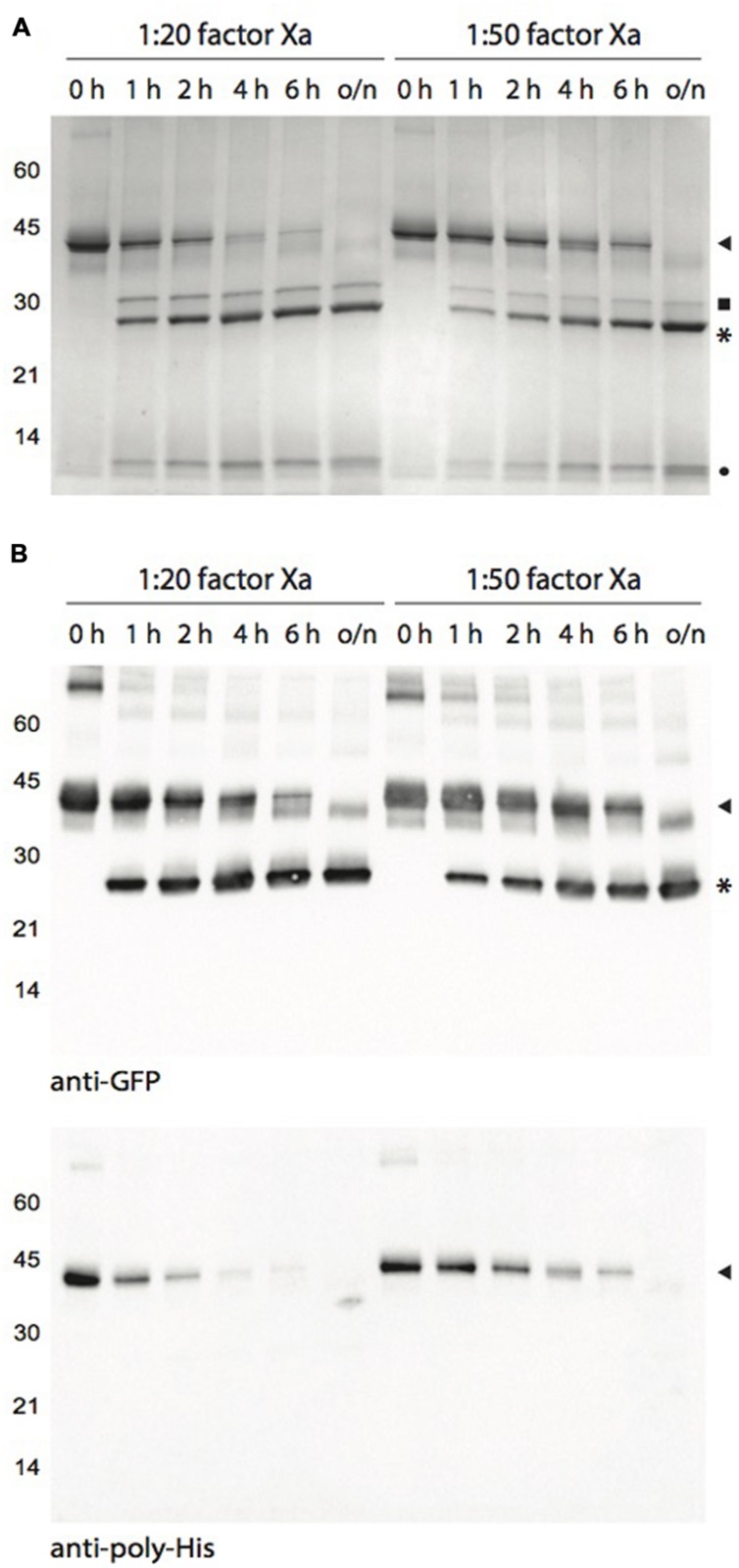
**Human factor X_a_-mediated cleavage of Cysta-tag–GFP at different incubation time points. (A)** Coomassie blue-stained polyacrylamide gel for Cysta-tag-GFP fusion samples incubated with factor X_a_ at two enzyme::protein ratios for 1, 2, 4, 6 h, or overnight (o/n). Digestion was monitored by immunoblotting with polyclonal primary antibodies directed against GFP or the poly-His motif **(B)**. Arrowheads point to the Cysta-tag–GFP fusion, asterisks (^∗^) to free GFP, closed circle to free Cysta-tag, and closed square to residual (contaminant) factor X_a_.

Initial attempts to digest factor X_a_- and enterokinase-cleavable Cysta-tag–α_1_AT fusions were not successful, likely due to either steric hindrance at the protease cleavage site or to the well-documented inhibitory effect of α_1_AT against several Ser proteases including factor X_a_ (Morrissey, 1998). To confirm utility of the Cysta-tag approach to produce proteins free of their affinity partner, we designed a factor X_a_-cleavable Cysta-tag fusion with GFP, also taking this opportunity to use a non-secreted version of the Cysta-tag to direct fusion protein accumulation in the cytosol. Cysta-tag–GFP was expressed in *N. benthamiana* leaves and purified as described above for the Cysta-tag–α_1_AT fusions (**Figure 5**). As expected, Coomassie blue-stained gels following SDS-PAGE (**Figure 5A**) and immunoblotting of both GFP and the Cysta-tag poly-His motif (**Figure 5B**) confirmed purification to high purity of a 38-kDa Cysta-tag–GFP fusion product, along with a product of higher molecular weight likely corresponding to Cysta-tag–GFP::GFP–Cysta-tag dimers as a result of GFP dimerization (Tsien, 1998). These observations demonstrate the potential of Cysta-tag-based expression for the affinity purification of recombinant proteins from leaf tissues regardless of their subcellular localization in transfected cells. They also show that the Cysta-tag could be detected with anti-poly-His antibodies (**Figure 5B**) and thus be used to monitor recombinant protein expression and purification processes.

Enzymatic removal of the Cysta-tag was performed with molar ratios of factor X_a_ fixed at 1:20 (as suggested by the manufacturer) and 1:50 relative to the recombinant protein (**Figure 5**). Both protease concentrations allowed for an effective cleavage of the fusion, readily reducing the amount of intact 38-kDa fusion protein –and dismantling the GFP fusion dimers– to generate a 27-kDa protein corresponding to free GFP. Cleavage at the 1:20 ratio was nearly complete after 4 h, confirming efficient removal of the affinity tag under standard processing conditions. The 1:50 ratio required an overnight incubation but the cleavage was also complete, pointing to the possibility of minimizing protein sample contamination with residual factor X_a_ after enzymatic cleavage for those proteins that support long incubation periods.

## Conclusion

Our goal in this study was to devise a protease-removable fusion tag for the IMAC purification of plant-made proteins in native conditions. Building upon our finding that tomato cystatin SlCYS8 can act as a stabilizing fusion partner for secreted proteins in plant leaf biofactories (Sainsbury et al., 2013), we engineered a chimeric tag for IMAC that is also detectable using readily available anti-poly-His antibodies and thus useful to monitor the expression and purification of recombinant proteins. Through molecular modeling we identified a physically unconstrained site for poly-His motif insertion, located in an exposed surface loop of SlCYS8 distal to both the inhibitory loops and N-terminus involved in protease inhibitory activity. The expression rate, overall stability and anti-papain potency of the resulting chimeric protein were unaltered compared to the parent protein, SlCYS8. This novel tag can be linked to a protein of interest using peptide linkers encoding different endoprotease cleavage sites. Protein purification using the Cysta-tag results in efficient and reproducible recovery of high-quality protein products, regardless of their subcellular localization *in planta*. Our results demonstrate the general usefulness of Cysta-tag fusions for recombinant protein purification from plant sources under mild, non-denaturing conditions. Indeed, a recent study reported the use of the Cysta-tag for the heterologous expression and purification of a difficult to express plant β-glucosidase in native conditions, permitting a first functional characterization of this previously intractable enzyme (Mageroy et al., 2015). We here developed the Cysta-tag for the purification of plant-made proteins, but the diversity of tools and protocols already available for poly-His-based IMAC in various expression systems make our new approach potentially applicable to any prokaryotic or eukaryotic host.

## Author Contributions

FS conceived the study with DM, took charge of the experimental design, performed lab experiments with PJ, and wrote a first draft of the manuscript. PJ contributed to the experimental design, performed lab experiments with FS, and contributed to the first draft of the manuscript. JV conceived the Cysta-tag with FS, performed the *in silico* (modeling) analyses, and contributed to the writing of the manuscript. M-CG contributed to the experimental design, coordinated the lab experiments, and contributed to the writing of the manuscript. DM conceived the study, contributed to the experimental design, coordinated the study, and prepared the last version of the manuscript.

## Conflict of Interest Statement

The authors declare that the research was conducted in the absence of any commercial or financial relationships that could be construed as a potential conflict of interest.
