# Supplementary material for: A Chimeric Affinity Tag for Efficient Expression and Chromatographic Purification of Heterologous Proteins from Plants
Source: Front Plant Sci. 2016 Feb 15;7:141. doi: 10.3389/fpls.2016.00141 (PMC4753422; doi:10.3389/fpls.2016.00141)
Supplement: Supplementary file 1 [file Image_1.PDF]

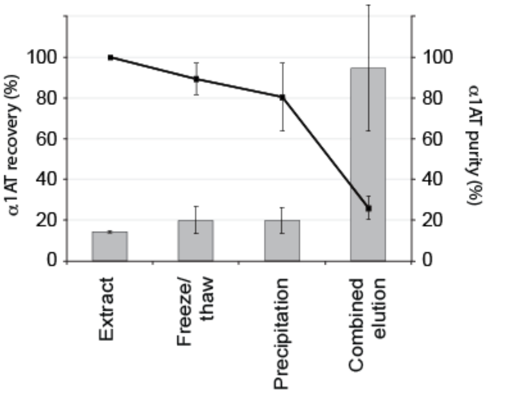

FIGURE S1 | IMAC purification of enterokinase-cleavable Cysta-tag–α_1AT fusion. Cysta-tag and α_1AT domains linked by the enterokinase-cleavable linker were transiently expressed in *N. benthamiana* leaves. α_1AT recovery (line) and purity (columns) rates during purification were calculated based on ELISA assays for α_1AT and leaf total soluble protein determinations. Data are mean values of three independent purification rounds ± SD.
